# Successful prednisolone or calcimimetic treatment of acquired hypocalciuric hypercalcemia caused by biased allosteric CaSR autoantibodies

**DOI:** 10.1172/jci.insight.156742

**Published:** 2022-10-24

**Authors:** Noriko Makita, Junichiro Sato, Katsunori Manaka, Kimiko Akahane, Takahiro Ito, Hajime Yamazaki, Akira Mizoguchi, Yusuke Hikima, Hirofumi Horikoshi, Masaomi Nangaku, Taroh Iiri

**Affiliations:** 1 Department of Nephrology and Endocrinology, The University of Tokyo Graduate School of Medicine, Tokyo, Japan.; 2 Department of Endocrinology and Metabolism, Tosei General Hospital, Aichi, Japan.; 3 Shirakabe Internal Clinic of Diabetes, Hypertension and Thyroid, Aichi, Japan.; 4 Department of Nephrology, Nagaoka Red Cross Hospital, Niigata, Japan.; 5 Department of Endocrinology and Diabetes, Ichinomiya Municipal Hospital, Aichi, Japan.; 6 Department of Pharmacology, St. Marianna University School of Medicine, Kanagawa, Japan.

**Keywords:** Autoimmunity, Endocrinology, Autoimmune diseases, G protein&ndash;coupled receptors, G proteins

## Abstract

Biased agonism is a frontier field in GPCR research. Acquired hypocalciuric hypercalcemia (AHH) is a rare disease caused by calcium-sensing receptor (CaSR) autoantibodies, to date, showing either simple blocking or biased properties (i.e., stimulatory or blocking effects on different downstream signaling pathways). This emphasizes the importance of the Gi/o (pertussis toxin–sensitive G proteins, whose βγ subunits activate multiple signals, including ERK1/2) in regulating parathyroid hormone secretion. We here describe 3 patients with symptomatic AHH who shared characteristics with the 2 cases we previously reported as follows: (a) elderly (74–87 years at diagnosis), (b) male, (c) unexpectedly showed no other autoimmune diseases, (d) showed spontaneously fluctuating Ca levels from approximately normal to near fatally high ranges, (e) acute exacerbations could be successfully treated with prednisolone and/or calcimimetics, (f) the presence of CaSR autoantibodies that operated as biased allosteric modulators of CaSR, and (g) were likely to be conformational (i.e., recognizing and, thereby, stabilizing a unique active conformation of CaSR that activates Gq/11, activating phosphatidylinositol turnover, but not Gi/o). Our observations with these prominent commonalities may provide new insights into the phenotype and characteristics of AHH and the mechanisms by which the biased agonism of GPCRs operate.

## Introduction

Biased agonism is a paradigm for explaining the selective activation of a certain and desirable signal through a GPCR that can typically mediate diverse signaling responses (1–9). The mechanisms by which selective signaling for the benefit of the cell is achieved via a “selectivity-loose” GPCR that activates multiple G protein/downstream signals remains a core question to be resolved in GPCR signaling research. An early hypothesis in this regard was that a specific combination of β and γ subunits enable a selective interaction between a specific GPCR and G protein. For example, it was shown that β3γ3 facilitates an interaction between the somatostatin SSTR2 receptor and Go2, a member of the pertussis toxin–sensitive (PTX-sensitive) Gi/o family of G proteins, while β1γ4 enables an interaction between muscarinic M4 receptor and Go1, another Gi/o family G protein (10, 11). An alternative theory has also been proposed in which a spatial regulation mechanism operates involving the specific colocalization of a GPCR and a G protein either at a certain site (i.e., lipid rafts or caveolae) within the plasma membrane or at another intracellular membrane to promote selective signal activation (12, 13).

A relatively recent model of selective activation through a GPCR is biased agonism (1–8). When a typical agonist activates a corresponding GPCR, multiple G proteins are often then activated, leading to multiple signal pathway responses. In contrast, a biased agonist (1, 2, 4, 6), or a combination of a usual (“selectivity-loose”) agonist and a biased allosteric modulator causes a unique active conformation of the GPCR (3, 5, 7–9, 14, 15) and the subsequent activation of a specific G protein/signaling mechanism only. Biased agonism has been sometimes described as just theoretical or often only in terms of pharmacological manipulation. Notably, however, we have now reported on the first series, to our knowledge, of autoantibodies against a calcium-sensing receptor (CaSR) that function as endogenous-biased allosteric modulators of this receptor to regulate 2 G protein signals, (i.e., Gq/11 and Gi/o signals in our case) in opposite directions (14, 15), though this is a pathophysiological phenomenon.

Autoantibodies against GPCRs can result in endocrine disease, though this is a rare occurrence (16–18). It is known, for example, that stimulatory autoantibodies against the thyroid-stimulating hormone (TSH) receptor cause Graves’ disease, while gain-of-function mutations of the TSH receptor cause familial hyperthyroidism. Blocking antibodies cause hypothyroidism, while loss-of-function mutations of the receptor can lead to congenital hypothyroidism (19, 20). In the case of CaSR (21–28), genetic diseases caused by its mutations and acquired diseases caused by autoantibodies directed against it have been described. Extracellular Ca levels are tightly controlled by CaSR expressed in parathyroid epithelial cells and at the basolateral membrane in cells of the thick ascending loop of Henle. When the extracellular Ca levels increase, increased Ca ions activate CaSR in the parathyroid gland, from which the secretion of parathyroid hormone (PTH) is inhibited. Inversely, when the extracellular Ca levels decrease, PTH secretion is upregulated. Gain-of-function mutations of CaSR stimulate its signaling pathways and inhibit PTH secretion from the parathyroid gland, resulting in hypocalcemia. This disorder is called autosomal dominant hypocalcemia 1 (ADH1) (27).

In the kidney, CaSR signaling activation in cells of the thick ascending loop of Henle results in hypercalciuria (23). Loss-of-function mutations of CaSR at 1 or both alleles inhibit CaSR signaling and cause increased PTH secretion, referred to as familial hypocalciuric hypercalcemia 1 (FHH1) and neonatal severe hyperparathyroidism (NSHPT), respectively (26, 27). Also in the kidney, a CaSR signaling block in cells of the thick ascending loop of Henle causes hypocalciuria.

As an acquired disease analogous to ADH1, acquired hypoparathyroidism, including immune-checkpoint inhibitor-related hypoparathyroidism, has been reported to be caused by activating autoantibodies against CaSR (29–34). Acquired hypocalciuric hypercalcemia (AHH), which is analogous to FHH1 or NSHPT, has been reported to be also caused by autoantibodies against CaSR (14, 15, 35–38). Depending on the reported AHH cases, autoantibodies work either as pure blocking entities (35) or in a biased manner (14, 15). By investigating their mode of action, both the pathophysiological mechanisms underlying this heterologous disease from the perspective of the epitopes detected and possible effective treatments and the physiological mechanisms by which PTH secretion is regulated in the human body have been further elucidated (15).

In our present study, we report on 3 additional patients with AHH who were elderly men with no evidence of any other autoimmune disorders such as chronic thyroiditis, thus, differing from most autoimmune cases. This was also the case for the 2 patients with AHH we had previously described. These 3 individuals displayed symptomatic PTH-dependent hypercalcemia with hypocalciuria and were, thus, clinically suspected of having AHH. This diagnosis was subsequently confirmed using in vitro analysis. Their Ca levels spontaneously fluctuated from approximately normal to subfatally high ranges. In instances of acute exacerbation, all 3 patients were successfully treated with prednisolone (PSL) and/or calcimimetics. Their CaSR autoantibodies operated as biased allosteric modulators of this receptor and were likely to be conformational (i.e., to recognize a unique conformation of CaSR).

The 3 additional patients with AHH analyzed in the present study provide further mechanistic insights into this rare disease with regard to the biased agonism of CaSR (i.e., biased allosteric modulation of this GPCR) and raise further questions about why the autoantibodies of some patients with AHH show unique characteristics.

## Results

### Patients with AHH and their clinical courses

#### Patient-1 (Pt-1).

**Figure 1:**
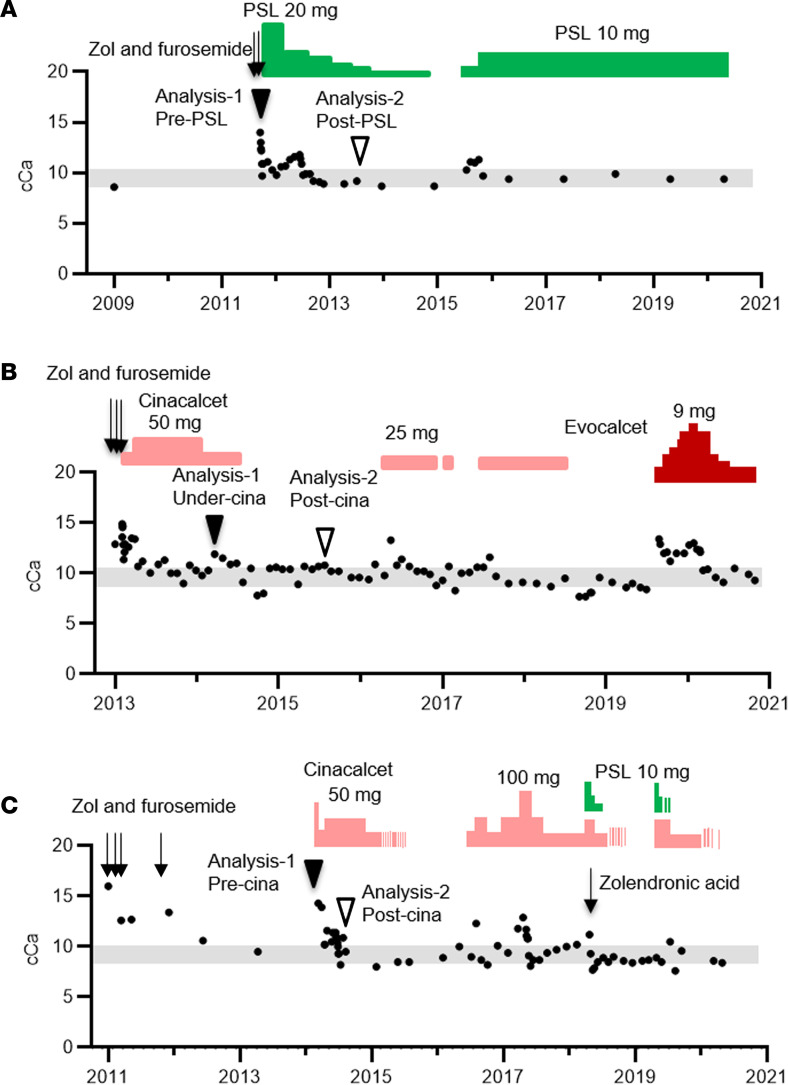
Temporal profile of the corrected serum Ca levels in the 3 study patients with AHH. (**A**) Pt-1, (**B**) Pt-2, and (**C**) Pt-3. In **A**–**C**, black filled circles denote the corrected Ca levels, gray bar indicates the reference interval of Ca level, and filled and open arrowheads indicate the timing of the in vitro analysis (Analysis-1 and Analysis-2). Zol, zolendronic acid.

The first of the study patients with AHH was a 76-year-old man with a chief complaint of appetite loss and in whom PTH-dependent hypercalcemia with hypocalciuria was detected (cCa, 13.8 mg/dL; iP, 1.9 mg/dL; Cre, 0.82 mg/dL; intact PTH, 60 pg/mL; and FECa of 0.32%; Table 1). More than 3 years prior to his presentation at our clinic, his serum Ca level was normal (cCa 8.6 mg/dL, iP 3.6 mg/dL). He had been treated at that time for bronchial asthma as an allergic disease. He had neither a familial history of hypercalcemia nor any personal history of autoimmune disease. He harbored no mutations in the *CASR*, *GNA11*, and *AP2S1* genes. Despite undergoing a conservative treatment regimen with furosemide (10 mg/d) and zolendronic acid (4 mg/month), after an emergent therapy with hydration and elcatonin, he still showed hypercalcemia (cCa, 11.8 mg/dL; and FECa, 0.22%). He was then administered PSL at 20 mg/d, and a diagnosis of AHH was simultaneously confirmed by in vitro analysis. After 1 month, his Ca level had normalized (Ca, 10.0 mg/dL; and FECa, 0.67%), and the PSL treatments were gradually tapered off. Within 1 year, however, his Ca level had increased again, and a PSL treatment was recommenced at 5 mg per day and has been maintained at 10 mg per day since (Figure 1A).

#### Patient-2 (Pt-2).

The next AHH case was a 75-year-old man, also with a chief complaint of appetite loss and who had PTH-dependent hypercalcemia with hypocalciuria (cCa, 13.6 mg/dL; iP, 2.2 mg/dL; Cre, 0.84 mg/dL; intact PTH, 101 pg/mL; and FECa, 0.04%; Table 1). Two years prior to this, his Ca level had been normal (cCa, 10.4 mg/dL). He had undergone a prostatectomy due to prostate cancer and had neither a family history of hypercalcemia nor personal history of autoimmune disease. He had no mutations in the *CASR*, *GNA11*, and *AP2S1* genes. AHH was suspected and treatment with cinacalcet (25 mg/d) was commenced after emergent therapy with hydration, furosemide, and zolendronic acid. At a higher cinacalcet treatment of 50 mg/d, his Ca levels became permissive, and the dose was, thus, reduced to 25 mg/d. His Ca levels then rebounded to a high concentration and AHH was subsequently confirmed by in vitro analysis. After 6 months, he showed hypocalcemia and his cinacalcet therapy was discontinued. This was followed by the onset of permissive hypercalcemia. At approximately 1 year after his treatment was ceased, his Ca levels flared up again and the cinacalcet was recommenced. He had since shown intermittent hypocalcemia followed by the cessation of his cinacalcet or evocalcet regimens, and his Ca levels have been well controlled using these treatment approaches (Figure 1B).

#### Patient-3 (Pt-3).

The third patient with AHH was an 87-year-old man with a disturbed consciousness in whom PTH-dependent hypercalcemia with hypocalciuria was revealed (cCa, 14.3 mg/dL; iP, 2.2 mg/dL; Cre, 0.97 mg/dL; intact PTH, 32 pg/mL; and FECa, 0.18%; Table 1). He had been suspected 3 years previously of having primary hyperparathyroidism of unknown localization. He had a history of rectal cancer, which had been treated with a colectomy followed by irradiation for positive surgical margin 6 years prior to his current presentation. He had no family history of hypercalcemia nor personal history of autoimmune disease. Following treatment with intermittent hydration, elcatonin, and zolendronic acid with furosemide for about 2 years, his Ca levels normalized without further intervention for 1 year. His hypercalcemia eventually recurred, however, and we confirmed a diagnosis of AHH by in vitro analysis. At that time, he showed no evidence of rectal cancer recurrence. He was then initially administered cinacalcet at 25 mg/d with an eventual dosage increase up to 75 mg/d after emergent therapy with hydration, furosemide, and zoledronic acid. After a maintenance therapy with cinacalcet at 50 mg/d, he again showed hypocalcemia, and this treatment was stopped. He developed hypercalcemia once more 10 months later, and a cinacalcet regimen was recommenced. Despite receiving a high dosage (100 mg) of cinacalcet at that time, his Ca levels were not controlled, and PSL was added to the treatment for a short period. After repeated similar episodes, he passed away due to intestinal bleeding caused by a recurrence of his rectal cancer 6 years after the diagnosis of AHH (Figure 1C).

### Serum reactivity in the 3 patients with AHH to COS-7 or HEK293 cells expressing CaSR

**Figure 2:**
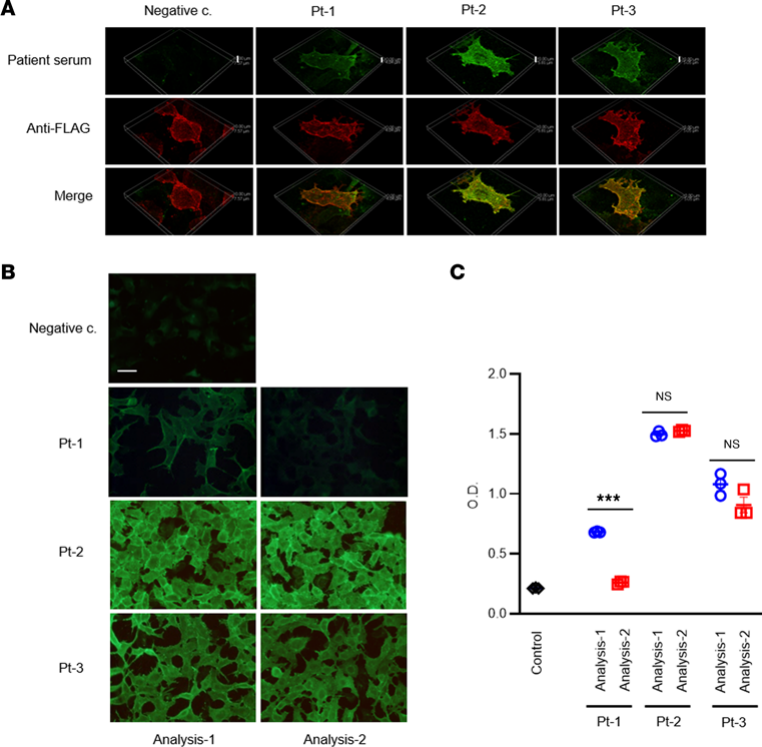
Immunofluorescence and cell surface ELISA using serum of patient with AHH against COS-7 cells and HEK293 cells expressing human CaSR. (**A**) Immunofluorescence staining of COS-7 cells transiently expressing FLAG-tagged human CaSR with the sera of the patient with AHH/control (1:100) and anti-FLAG antibody (1:2,000). Scale bar: negative c., 7.57 mm; Pt-1, 4.04 mm; Pt-2, 5.8 mm; and Pt-3, 5.05 mm. (**B**) Immunofluorescence staining of HEK293 cells expressing human CaSR with the sera of patient with AHH/control (1:100). Scale bar: 50 mm. (**C**) Cell surface ELISA against HEK293 cells expressing human CaSR with the sera of patient with AHH/control (1:500). Statistical analysis was performed using a 2-sided Student’s *t* test. Values represent the mean ± SEM of triplicate determinations. Each set of results is representative of at least 2 additional experiments. ***P < 0.001.

Immunofluorescence analyses of the sera from all 3 patients with AHH using 3-dimensioned confocal microscopy (Figure 1, A–C, Analysis-1) indicated reactivity to COS-7 cells transiently expressing FLAG-tagged CaSR without permeabilization. This immunoreactivity of the patient serum samples to COS-7 cells was found to be almost completely colocalized with that of an anti-FLAG antibody (Figure 2A). These serum samples also reacted to HEK293 cells stably expressing CaSR (HEK293-CaSR cells) without permeabilization (Figure 2B). This reactivity was significantly reduced after PSL treatment in Pt-1 (Figure 1A, Analysis-2) but not after discontinuing cinacalcet treatment in Pt-2 (Figure 1B, Analysis-2) or commencing cinacalcet treatment in Pt-3 (Figure 1C, Analysis-2). These trends were confirmed by cell surface ELISA (Figure 2C).

### Effects of IgG of patients with AHH on Ca-stimulated inositol phosphate-1 accumulation and ERK1/2 phosphorylation

**Figure 3:**
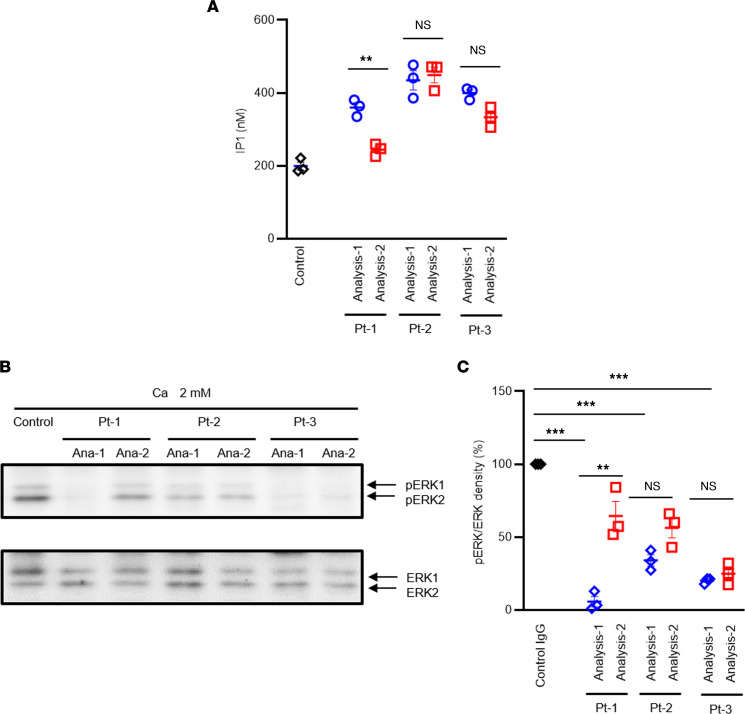
Ca-stimulated inositol 1-phosphate accumulation and phosphorylation of ERK1/2 following the coadministration of IgG of patient with AHH at 2 time points. HEK293 cells expressing human CaSR stably were stimulated by 2.0 mM of Ca with 2 mg/dL of IgG. (**A**) Inositol 1-phosphate accumulation was shown. (**B**) The phosphorylation of ERK1/2 (pERK) was detected by immunoblot (IB), and each ERK1/2 was detected by reblotting of the same membrane. (**C**) Intensity of pERK and ERK was analyzed by ImageJ software (NIH), and each intensity ratio (%) against the ratio at 2.0 mM Ca with control IgG was calculated, respectively. Statistical analysis was performed using 2-sided Student’s *t* test in **A** or Tukey’s multiple comparison test in **C**. Values represent the mean ± SEM of triplicate determinations. Each set of results is representative of at least 2 additional experiments. **P < 0.01, ***P < 0.001.

We investigated functional changes in the IgG of our patients with AHH at 2 time points using HEK293-CaSR cells, in the same manner described in our previous study (Figure 3). We previously reported using HEK293 cells expressing CaSR that CaSR-induced inositol monophosphate accumulation was mostly dependent on Gq/11 because an exogenously expressed regulator of G protein signaling (RGS) domain of GRK2 (14, 39), which captures activated Gqα, inhibited this signal (14, 15). We also reported that this signal was mostly independent of Gi/o, the PTX-sensitive G proteins, because treatment with PTX, which inhibits Gi/o signals, did not inhibit this signal (14, 15). At a Ca level of 2.0 mM, inositol 1-phosphate accumulation was found to be augmented by the coadministration of the IgG of each patient with AHH at the time of Analysis-1 (Figure 3A), which was similar to the findings in the 2 previous cases we reported (14, 15). After PSL treatment, Ca-stimulated inositol 1-phosphate accumulation decreased (Figure 3A) in Pt-1 (Figure 1A, Analysis-2). After discontinuing cinacalcet treatment (Figure 1B, Analysis-2) or commencing this treatment (Figure 1C, Analysis-2), Ca-stimulated inositol 1-phosphate accumulation continued to be higher than the control IgG in both Pt-2 and Pt-3 (Figure 3A). Ca-stimulated inositol 1-phosphate accumulation seemed to slightly decrease in Pt-3 (Figure 3A), although not significantly, possibly reflecting a fluctuation of autoimmunity in this case.

In contrast to the aforementioned findings, ERK1/2 phosphorylation at the Ca concentration of 2.0 mM was suppressed by the coadministration of the IgG of each patient with AHH at the timing of Analysis-1 (Figure 3, B and C, and Supplemental Figure 1; supplemental material available online with this article; https://doi.org/10.1172/jci.insight.156742DS1). After PSL treatment, this suppression was recovered at least partially in Pt-1 (Figure 1A, Analysis-2). After ceasing the cinacalcet treatment in Pt-2 (Figure 1B, Analysis-2) or after commencement of this therapy in Pt-3 (Figure 1C, Analysis-2), the suppression remained in both cases, although it appeared to have slightly recovered in Pt-3, consistent with the notion that cinacalcet does not affect autoimmunity.

### Ca-stimulated ERK1/2 phosphorylation and the effects of PTX and Gq inhibitors in HEK293 cells expressing CaSR

**Figure 4:**
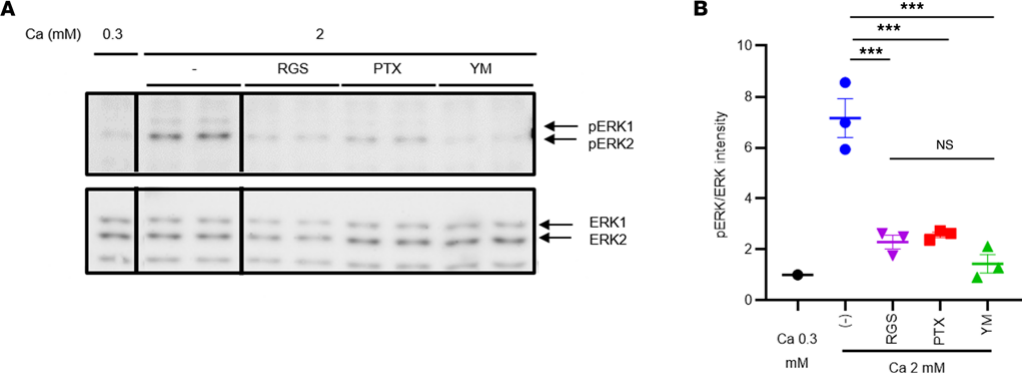
Ca-dependent pERK and the effects of PTX, YM, and RGS in HEK293 cells transiently expressing CaSR and RGS or a mock control. (**A**) Ca-dependent pERK and the effects of RGS, 500 ng/mL PTX for 4 hours or 10 nM YM were detected by IB, and each ERK1/2 was detected by reblotting of the same membrane. The lanes were run on the same gel but were noncontiguous. (**B**) Intensities of pERK and ERK were analyzed by ImageJ software, and each ratio against the ratio at 0.3 mM Ca was calculated, respectively. Statistical analysis was performed using a Tukey’s multiple comparison test. Values represent the mean ± SEM of triplicate determinations. Each set of results is representative of at least 2 additional experiments. ***P < 0.001.

**Figure 5:**
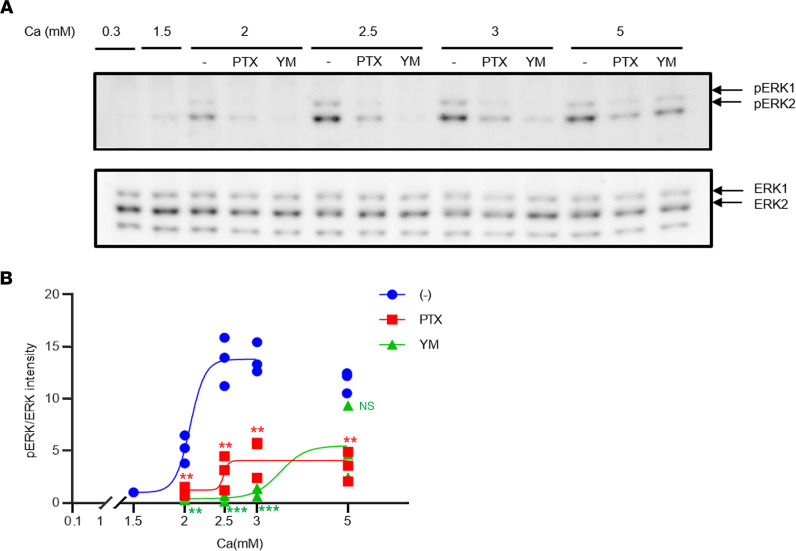
Ca-dependent pERK and the effects of PTX and YM in HEK293 cells stably expressing CaSR. (**A**) Ca-dependent pERK and the effects of pretreatment of 500 ng/mL PTX for 4 hours or 10 nM YM were detected by IB, and each ERK1/2 was detected by reblotting of the same membrane. (**B**) Intensities of pERK and ERK were analyzed using ImageJ software, and each ratio against the ratio at 1.5 mM Ca was calculated, respectively. Each point reflects a Ca-dependent ratio (blue filled circle; EC_50_, 2.06 ± 0.01 mM; and E_max_, 13.8, calculated except ratio at 5 mM Ca) under exposure to PTX (red filled square), and YM (green filled triangle). Statistical analysis was performed using a Tukey’s multiple comparison test. Values represent the mean ± SEM of triplicate determinations. Each set of results is representative of at least 2 additional experiments. **P < 0.01, ***P < 0.001.

**Figure 6:**
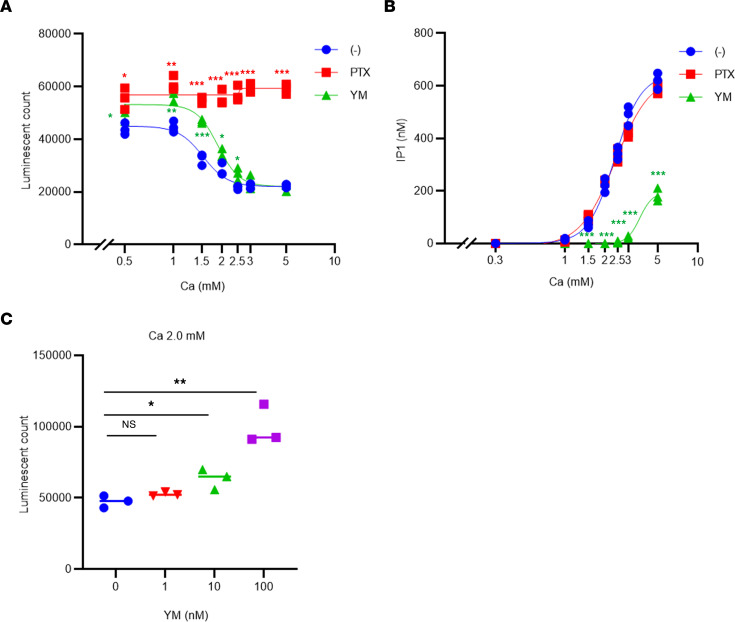
Ca-dependent Gi/o-cAMP inhibition and Gq/11-inositol 1-phosphate accumulation with the effects of PTX and YM in HEK293 cells stably expressing CaSR. (**A**) A Ca-dependent luminescence count stimulated by 3 μM forskolin (blue filled circle; IC_50_, 1.52 ± 0.09 mM) with the effects of 500 ng/mL PTX for 4 hours (red filled square) and 10 nM YM (green filled triangle; IC_50_, 1.88 ± 0.03 mM). (**B**) Ca-dependent inositol 1-phosphate accumulation (blue filled circle; EC_50_, 2.37 ± 0.02 mM; and E_max_, 644 nM) with the effects of 500 ng/mL PTX for 4 hours (red filled square) and 10 nM YM (green filled triangle). (**C**) Luminescence count stimulated by 2.0 mM Ca and 3 μM forskolin without (blue filled circle), or with 1 nM (red filled triangle), 10 nM (green filled triangle), or 100 nM (square filled purple) YM. Statistical analysis was performed using a 2-sided Student’s *t* test (**A** and **B**) or Tukey’s multiple comparison tests (**C**). Values represent the mean ± SEM of triplicate determinations. Each set of results is representative of at least 2 additional experiments. *P < 0.05, **P < 0.01, ***P < 0.001.

We have also previously reported that, at a Ca level of up to 2.5 mM, ERK1/2 phosphorylation is largely dependent on the Gi/o proteins and their βγ subunits because both PTX treatment and exogenously expressed Gtα, which captures free βγ subunits, largely inhibited CaSR-dependent ERK1/2 phosphorylation in HEK293 cells expressing CaSR stably (HEK293-CaSR cells) (14). We next confirmed that EKR1/2 phosphorylation was largely dependent on the Gi/o proteins in HEK293-CaSR cells, based on the effect of PTX, which was consistent with our previously reported findings (14) (Figure 4, A and B, and Figure 5, A and B). Recently, however, it has been reported that some Giβγ-induced signals leading to Ca mobilization also depend on Gq (40). Therefore, we queried whether CaSR-induced ERK1/2 phosphorylation was also dependent on Gq/11 using an exogenously expressed RGS (14, 39) and also the YM-254890 (YM) (41), both of which have been reported to specifically inhibit the Gq/11 signal. Unexpectedly, both RGS and YM largely inhibited Ca-dependent ERK1/2 phosphorylation at a concentration of less than 2.5 mM (Figure 4, A and B, and Figure 5, A and B). However, the specificity of YM remains a matter of dispute (42). In the system we used to investigate Gi activation through the Ca-dependent inhibition of cAMP accumulation stimulated by forskolin, PTX treatment almost completely inhibited the Gi-dependent inhibition of cAMP (Figure 6A). A 10 nM concentration of YM also inhibited the Gi-dependent inhibition of cAMP, at least in part, especially at a nonsaturated concentration of Ca (Figure 6A). YM inhibited inositol 1-phosphate accumulation at the Ca concentration of less than 3.0 mM (Figure 6B). The inhibitory effects of YM upon Gi were found to be dose dependent (Figure 6C). Unfortunately, however, the inhibition of the Gi signal using the patient’s IgG could not be evaluated in this cAMP assay so far because IgG appeared to have nonspecific effects and because the amount of our patient’s IgG is limited.

Taken together, we found from these analyses that, at least at less than 2.5 mM Ca, CaSR-dependent ERK1/2 phosphorylation is likely to be dependent on both Gi/o and Gq/11. In addition, a potentially greater suppression of ERK phosphorylation by YM may be due to its inhibition of not only Gq/11 but also Gi/o proteins, at least in part. Based on these results, it was confirmed that the IgG of patients with AHH likely inhibits Gi/o and stimulates Gq/11, as we previously reported (14, 15).

### Serum reactivity in patients with AHH against the extracellular domain of CaSR and its mutants harboring alanine substitutions at aa 214–235

**Figure 7:**
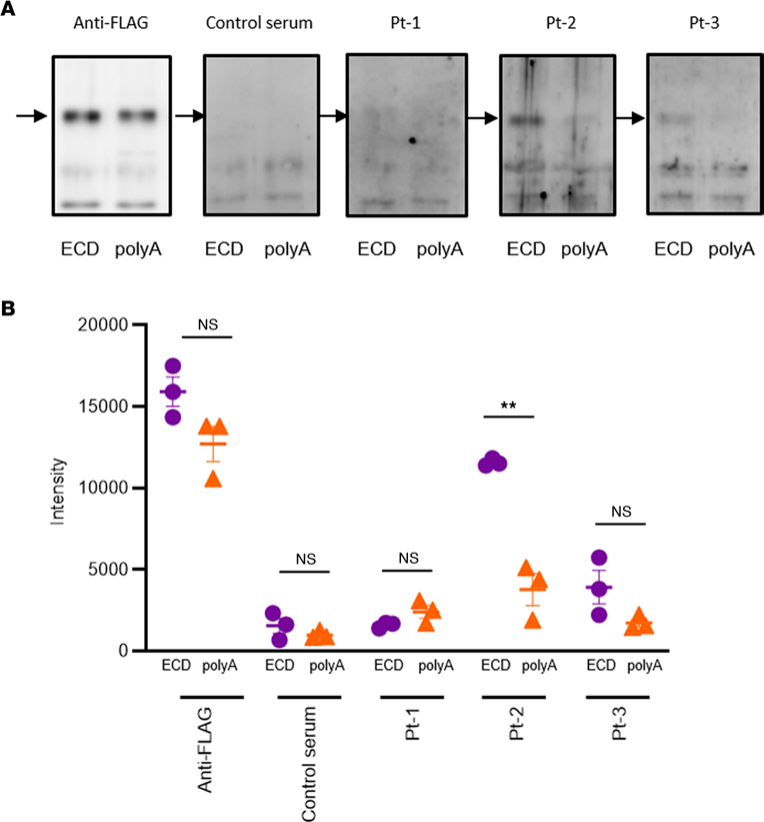
IB analysis of serum of study patient with AHH reactivity against the CaSR-ECD or an ECD mutant containing alanine substitutions at aa 214–235 (polyA). (**A**) IP ECD or polyA was resolved by SDS-PAGE and IB against anti-FLAG (1:5,000) or sera from a negative control and the 3 patients with AHH (1:200). (**B**) Bands were analyzed by ImageJ software. Statistical analysis was performed using a 2-sided Student’s *t* test. Values represent the mean ± SEM of triplicate determinations. Each set of results is representative of at least 2 additional experiments. **P < 0.01. Purple filled circles, ECD; orange filled triangles, polyA.

Immunofluorescence analysis and cell surface ELISA using HEK293-CaSR cells confirmed that the sera of our 3 current patients with AHH reacted against the extracellular domain of CaSR (CaSR-ECD) We previously reported that the autoantibodies of 2 patients with AHH recognized the conformation of aa 214–235 within the CaSR-ECD (15). Thus, we investigated whether the sera of our current cases of AHH might recognize this same sequence of residues (Figure 7, A and B). The serum of Pt-2 reacted to the full-length CaSR-ECD, and this reactivity decreased against an ECD mutant in which residues 214–235 were substituted for alanine (CaSR-polyA). This result indicated that this serum likely recognizes this region of the ECD, at least in part. No particular band was detected against CaSR-ECD in Pt-1, while this reaction was detected, although not significantly, in Pt-3 (Figure 7, A and B). These findings were compatible with our earlier immunofluorescence and cell surface ELISA results (Figure 2, A–C), showing that the reactivities of Pt-1 and Pt-3 sera against COS-7 cells and HEK293 cells expressing CaSR were lower than those of Pt-2.

### Serum reactivity of patient with AHH against peptides covering the CaSR-ECD

**Figure 8:**
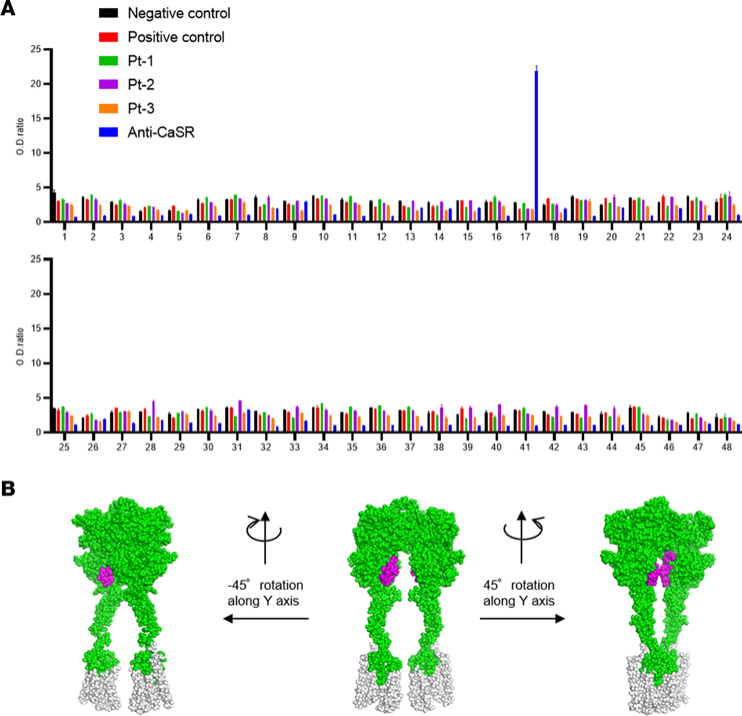
Peptide ELISA with patient/control sera against peptides almost covering the CaSR-ECD and a 3D representation of dimerized CaSR drawn using PyMOL. (**A**) Immunoreactivity against peptides covering the ECD or extracellular loop of CaSR. Each number reflects each peptide: 1: 1–20; 2: 21–31; 3: 32–43; 4: 44–58; 5: 56–70; 6: 70–79; 7: 79–95; 8: 96–110; 9: 108–122; 10: 123–137; 11: 138–150; 12: 151–164; 13: 165–179; 14: 177–191; 15: 187–203; 16: 204–213; 17: 214–238; 18: 239–255; 19: 256–266; 20: 267–281; 21: 282–292; 22: 293–307; 23: 308–314; 24: 314–323; 25: 322–329; 26: 330–343; 27: 344–358; 28: 359–373; 29: 374–391; 30: 392–405; 31: 406–420; 32: 419–428; 33: 428–440; 34: 441–455; 35: 456–470; 36: 471–483; 37: 484–493; 38: 494–508; 39: 506–520; 40: 519–532; 41: 533–546; 42: 547–561; 43: 559–573; 44: 571–585; 45: 586–603; 46: 667–686); 47: 724–773; and 48: 824–841. Values represent the mean ± SEM of triplicate determinations. Each set of results is representative of at least 2 additional experiments. (**B**) 3D depiction of dimerized CaSR based on 7DTW in the protein data bank drawn using PyMOL 2.0.7. Green, regions covered by the peptides cover; magenta, 214–235 residues of CaSR.

There have been some prior reports that the serum from patients with AHH or autoimmune hypoparathyroidism reacts to certain peptides that match segments of the CaSR-ECD (29, 31, 33–36, 38). In addition to those peptides, we prepared others to cover the entire portion of the ECD. We also prepared peptides constituting the extracellular loop of the CaSR (Figure 8, A and B). Our current sera of the patients with AHH did not react to any of these peptides, however, including those covering aa 214–235 (Figure 8A), suggesting that the autoantibodies in these cases may only recognize a specific conformation of the CaSR-ECD.

## Discussion

We here report on 3 patients with symptomatic AHH who harbor CaSR autoantibodies and who showed similar characteristics to 2 prior patients who we described (14, 15), i.e., (a) elderly, 74–87 years old at diagnosis; (b) male; (c) unexpectedly showing no evidence of other autoimmune diseases, which is atypical for this disorder (29, 36, 37); (d) spontaneously fluctuating serum Ca levels between a permissive to normal and high near-fatal range; (e) had been successfully treated with either PSL and/or cinacalcet when needed; (f) harbored CaSR autoantibodies operating as biased allosteric modulators of CaSR; and (g) these autoantibodies were likely to be conformational (i.e., to recognize and thereby stabilize a unique active conformation of CaSR that activates Gq/11 but not Gi/o).

Patients with AHH were originally reported as familial females showing asymptomatic hypercalcemia with relative hypocalciuria, a condition that is very similar to typical FHH1 (35). FHH1 caused by a heterologous loss-of-function mutation of CaSR is usually an asymptomatic disease that does not require treatment. In contrast, NSHPT, caused by a homozygous loss-of-function mutation of CaSR, is a very severe disorder that requires resection of all parathyroid glands immediately after birth (23). Based on its terminological analogy with FHH1, AHH may be misunderstood as being similar to FHH1. However, the severity of AHH depends on the titer of the autoantibodies against CaSR, such that it can range from asymptomatic like FHH1 to severely symptomatic like NSHPT (15). Moreover, during the clinical course of AHH, the disease status can fluctuate depending on the autoantibody titer of the patient (15). Although severe and symptomatic instances of AHH need to be treated, only a few such cases have been reported to date (15, 36).

High levels of extracellular Ca inhibit the secretion of PTH via the CaSR that activates at least Gq/11 and also Gi/o proteins. However, the molecular mechanism of this remains to be fully understood. It has been postulated that CaSR classically functions via Gq/11 (14, 21, 43). In support of this contention, a loss-of-function mutation of G11 causes FHH2 (27, 44), which is similar to FHH1, due to a disruption of CaSR activity. Conversely, a gain-of-function mutation of G11 causes ADH2 (27, 44, 45), which is similar to ADH1 due to a gain-of-function mutation of CaSR. In contrast, we (14, 15) and others (21) have proposed that not only Gq/11 but also Gi/o factors may play an additional important role in inhibiting PTH secretion. In our model system using HEK293 cells expressing CaSR, we have disclosed that CaSR autoantibodies work as biased allosteric modulators that stimulate Gq/11-phosphatidylinositol turnover and inhibit Gi/o-ERK1/2 phosphorylation (14, 15). We observed similar results in the current 3 AHH cases. Pallais et al. have reported that some CaSR autoantibodies only inhibit ERK1/2 phosphorylation in AHH (37). However, additionally, and unexpectedly, our current analyses revealed that ERK1/2 phosphorylation via CaSR at a Ca concentration of 2.0 mM is largely inhibited not only by PTX that inhibits GPCR-dependent activation of Gi/o, but also by Gq inhibitors, i.e., the RGS or YM. ERK1/2 phosphorylation (at 2.0 mM Ca) via CaSR might, therefore, be dependent on both the Gi/o and Gq/11. Recently, it has been reported that Giβγ-induced Ca mobilization also depends on Gq/11 (40). It has also been demonstrated that Gqα and Giβγ synergistically activate PLCβ3 and Ca mobilization (46). These results suggest, but do not yet prove, that the Gq/11 signal and Gi/o signal may synergistically stimulate ERK1/2 phosphorylation in our HEK293-CaSR cells. Because AHH autoantibodies augment Gq/11 signaling, we speculate that an inhibition of ERK1/2 phosphorylation by AHH autoantibodies may be caused by the suppression of Gi/o signaling, overcoming the potentiated Gq/11 signaling. Unfortunately, however, the inhibition of the Gi (but not the Go) signal by AHH autoantibodies could not be evaluated in the cAMP assay so far partly because the amount of our patient IgG is limited. A more direct demonstration of the effects of autoantibodies on Gi/o signaling will be desirable in the future. Furthermore, these results lead us to speculate that, in parathyroid cells as well, the Gq/11 signal and Gi/o signal may act synergistically to decrease PTH secretion. If our speculation is correct, inhibition of the Gq/11 signal or Gi/o signal may increase PTH secretion in parathyroid cells, resulting in the phenotype of AHH/FHH. And in our cases with CaSR autoantibodies working as biased allosteric modulators, we speculate that the inhibition of the Gi/o signal may have overcome the potentiated Gq/11 signal, overall resulting in our AHH phenotype.

There are 2 prior AHH cases that have been described in which PSL treatments were attempted. Of these patients, 1 showed improvement following this therapy (36), but no benefit was observed in the other case (37). In the report on the PSL-responsive case, however, the function of the autoantibodies was not analyzed. Our current study is, thus, to our knowledge, the first to describe the successful treatment of a conclusively diagnosed AHH case with PSL. Our analyses indicated that both the titer and the function of the CaSR autoantibodies had improved, mostly in parallel, in Pt-1. The autoantibody titer was found in this case to have decreased by immunofluorescence staining and ELISA after PSL treatment (Figure 2, B and C). With regard to the function of these CaSR autoantibodies, we further found that the inhibition of Ca-stimulated ERK1/2 phosphorylation using patient IgG was significantly recovered by PSL treatment (Figure 3, B and C). Notably, as Gi/o-dependent signaling is a key component of the regulation of PTH secretion (14, 37), this functional change is consistent with the observed clinical course in our present cases of AHH. Conversely, the augmentation of Ca-stimulated inositol 1-phosphate accumulation was found in our current analyses to be decreased by PSL treatment (Figure 3A).

In terms of the impacts of calcimimetic treatment, we have recently described the first case of AHH that was successfully treated with cinacalcet over a long period (15). We analyzed 2 additional cases being treated in our current study. Both patients showed fluctuation of their Ca levels with intermittent hypocalcemia, followed by a discontinuation of the cinacalcet therapy. Overall, the Ca levels in these 2 cases were well controlled. In Pt-2, we anticipated that in vitro analyses using serum sampled during the cinacalcet treatment (Figure 1B, Analysis-1) and then after this treatment had ceased (Figure 1B, Analysis-2) would reflect the observed clinical course. However, no particular differences were evident between these sets of assay data (Figure 3, A–C). During the period after the cinacalcet therapy had been discontinued, this patient showed slight hypercalcemia, indicating that CaSR autoantibodies continued to be produced. We speculated, therefore, that a slight change in the autoantibody titer might not be detectable by our analysis.

The autoantibodies detected in our current patients were found to function as biased allosteric modulators in a similar manner to our previously reported cases (14, 15) and differing from other cases (29). The observed mode of action in our current patients with AHH represents additional evidence that biased agonism operates endogenously in the human body and is not just a pharmacological phenomenon. In addition, the biased effects of our patients’ autoantibodies in relation to regulating 2 G protein signals in opposite directions further supports the importance of the Gi/o proteins in regulating PTH secretion.

Some of the significant questions arising from our present observations include where and how these autoantibodies react against CaSR. In the patients with AHH or autoimmune hypoparathyroidism reported by other researchers, the patient sera were found to be reactive against several peptides that can be found in the CaSR-ECD (peptides 41–69; 114–126; 171–195; 214–236; 344–358; and 374–391) (29, 31, 33–36, 38). The 114–126 peptide is particularly noteworthy in this regard, as both activating and blocking antibodies have been reported (31, 38). In contrast, the sera of our present AHH cases did not show any reactivity against these ECD peptides, including the 214–238 peptide. This suggests that the autoantibodies produced in our current cases may be conformational (i.e., they recognize a unique conformation of CaSR). Monoclonal antibodies derived from patients with AHH will play a key role in the future in elucidating where and how these autoantibodies react. A functional assessment of how a biased monoclonal antibody might stabilize a unique CaSR structure will likely provide insights into the mechanism of the specific GPCR signaling activation in AHH and assist with the development of drugs that will not have undesirable side effects.

It is uncertain why all of published research to date on CaSR autoantibodies that work as biased allosteric modulators has been limited almost exclusively to Japan. It is possible that this unique and somewhat complex signaling mechanism has simply been overlooked in some other studies. Alternatively, a causative genetic background that is unique to Japanese patients with AHH may be involved. This latter hypothesis is also compatible with our observation that autoantibodies in our AHH patients developed without other autoimmune diseases. Further investigation of this phenomenon represents an important future research question. Clarification of a possible underlying genetic background for our AHH cases may provide novel insights into the molecular mechanism of AHH itself and of the biased agonism of GPCRs in general.

In conclusion, we report here on 3 Japanese patients with AHH who shared unique clinical and in vitro properties. Our observations may help foster new insights into the phenotypes and characteristics of AHH, including the underlying genetic background and mechanisms of how the biased agonism of GPCRs operate.

## Methods

### Sample preparation.

Serum samples were collected and stored at –80° until just before use. Purified IgG was isolated using a Montage antibody purification kit (MilliporeSigma) followed by concentration using Amicon Ultra 15 (MilliporeSigma) for immediate assay (14, 15). Experiments using patient samples were performed under approval of the Institutional Review Board of the University of Tokyo.

### Chemicals.

We purchased all chemicals, including YM and PTX, from Wako Pure Chemical Industries (Fujifilm).

### Expression constructs, cell culture, and transfection.

Plasmid expressing only CaSR-ECD (aa 1–603) in pcDNA3.1 was made by subcloning PCR product using FLAG-tagged human CaSR in pcDNA3.1 as a template. Mutagenesis of CaSR-ECD replaced aa of 214–235 to alanines (CaSR-polyA) was performed by KOD -Plus- Mutagenesis Kit (TOYOBO) (15). Plasmid-expressing RGS was gifted by Tohru Kozasa (Department of Biochemistry, Yokohama University of Pharmacy, Yokohama, Japan) (14). HEK293 cells or COS-7 cells (both from Henry Bourne Lab, Department of Cellular and Molecular Pharmacology, UCSF, San Francisco, California, USA), maintained in DMEM containing 10% FBS, were transfected with constructs encoding CaSR, RGS, pGloSensor-22F cAMP plasmid (Promega Corporation), CaSR-ECD, or CaSR-polyA, using Lipofectamine 2000 (Thermo Fisher Scientific) (14, 47, 48). The transiently transfected cells were used for IP study, immunostaining study, cAMP assay, and ERK1/2 phosphorylation assay after 48 hours in culture. Otherwise, we used HEK293 cells stably expressing human CaSR, selected and cloned in medium containing 0.8 mg/mL G418 as described previously (14, 49–52).

### Immunofluorescence imaging.

Immunofluorescence imaging was performed using patient or control sera (1:100 dilution) (Figure 2, A and B). Briefly, HEK293 cells stably expressing human CaSR or COS-7 cells transiently expressing FLAG-tagged human CaSR were incubated with patient or control sera (1:100) and/or monoclonal anti-FLAG antibody (1:2,000) (catalog F3165, Sigma-Aldrich) for 1 hour at 37°C and fixed at 4°C for 15 minutes in 4% paraformaldehyde/PBS. Bound IgG were detected by using goat antihuman IgG tagged with Alexa Fluor 488 (green) (catalog A-11013, Molecular Probes) and antimouse IgG tagged with Alexa Fluor 568 (red) (catalog A-11004, Molecular Probes) (14, 53). Fluorescence images were collected by using confocal microscope AX or ECLIPSE E600 microscope (NIKON).

### Cell surface ELISA.

Immunoreactivity was quantified by cell surface ELISA. Briefly, HEK293 cells expressing human CaSR stably were plated onto 48-well plates. The next day, the cells were incubated with patient or control sera (1:500) for 1 hour at 37°C. After washing, the cells were fixed at 4°C for 15 minutes in 4% formaldehyde/PBS (without permeabilization). The cells were then incubated in HRP-conjugated antihuman IgG (catalog 074-1002, Kirkegaard and Perry Laboratories) (1:5,000) at room temperature for 1 hour. After washing, the cells were treated with substrates (*o*-phenylenediamine dihydrochloride, MilliporeSigma) for 5 minutes at room temperature. This reaction was stopped by the addition of an equivalent volume of 2.5 N HCl, and the absorption levels were read at 492 nm using a plate reader (EnSpire, PerkinElmer).

### Measurement of CaSR-stimulated inositol 1-phosphate accumulation.

Inositol 1-phosphate was measured by modified manufacturer’s protocol using inositol 1-phosphate homogeneous time resolved fluorescence kit (Cisbio). Briefly, HEK293 cells expressing the human CaSR were applied onto 96-well plates on the day before assay, preincubated with or without 10 nM YM for 30 minutes or PTX for 4 hours, and incubated with various concentrations of Ca or purified IgG at Ca 2.0 mM with 50 mM LiCl for 1 hour at 37°C. Then, the reaction was stopped by replacing and extracted by HBSS containing 1% Triton X, and inositol 1-phosphate was measured based on manufacturer’s procedure using homogeneous time resolved fluorescence technology. Calculated inositol 1-phosphate values based on standard curve were fitted to a 4-parameter sigmoidal concentration-response curve using Prism 8 software (GraphPad), and the values for EC_50_ were calculated from the curve.

### Measurement of cAMP-dependent luminescence signal.

HEK293-CaSR cells transfected with pGloSensor-22F cAMP plasmid 1 day before with or without PTX treatment for 4 hours were detached, centrifuged, and suspended with HBSS containing 5 mM HEPES (pH 7.4), 0.01% (w/v) BSA, 0.5 mM IBMX (Sigma-Aldrich), and 2 mM d-luciferin. Cells (600/μL) were seeded in a 384-well white microplate at a volume of 15 μL per well and incubated at room temperature for 2 hours in the dark with or without 5 μL of 5× YM for 30 minutes. A total of 5 μL of 5× Ca diluted in 15 μM forskolin were manually added to the cell and incubated at room temperature for 20 minutes. Measured luminescence signals were fitted to a 4-parameter sigmoidal concentration-response curve using Prism 8 software (GraphPad Prism) and the values for IC_50_ were calculated from the curve.

### Measurement of ERK1/2 phosphorylation.

For determining ERK1/2 phosphorylation, the cells were plated and starved for 12–16 hours in DMEM with 0.3 mM CaCl_2_. The cells were then stimulated with various concentrations of Ca with or without YM or purified IgG at Ca 2.0 mM for 2 hours at 37°C, and the reactions were terminated. Protein extracts were then analyzed on Western blotting using anti-phosphorylated ERK1/2 antibody (1:2,000) (E10) (catalog 9106, Cell Signaling Technology). Each membrane was stripped by stripping buffer (0.2 M glycine with 0.1% SDS, and 1% Tween 20, pH 2.2); washed by PBS and PBS-0.1% Tween twice, respectively, blocked; and incubated by anti-ERK1/2 (1:2,000) (Enzymology). The intensity of phosphorylated ERK1/2 and ERK1/2 was analyzed by ImageJ software, and each ratio against control ratio was calculated, respectively. Calculated signals were fitted to a 4-parameter sigmoidal concentration-response curve using Prism 8 software (GraphPad Prism) and the values for EC_50_ were calculated from the curve.

### IP.

The COS-7 cells transiently transfected CaSR-ECD or CaSR-polyA 48 hours before they were washed with ice-cold PBS and lysed in ice-cold lysis buffer (40 mM Tris-HCl [pH 7.4]; 100 mM NaCl; 2 mM MgCl_2_; 1% Triton X; 100 mM EGTA; and protease inhibitors) on ice by gently shaking each for 5 minutes. After 60 minutes, cell lysates were collected in tubes and centrifuged at 20,000*g* for 10 minutes at 4°C. Supernatants were incubated with 2 μg monoclonal anti-FLAG antibody (catalog F3165, Sigma-Aldrich) for 2 hours at 4°C and absorbed to protein G plus Sepharose (catalog sc-2002, Santa Cruz Biotechnology) for 1 hour at 4°C. Bound complexes were washed 3 times with IP buffer. Proteins were then analyzed on Western blotting using monoclonal anti-FLAG antibody (1:5,000) (catalog F3165, Sigma-Aldrich) and patients’ sera (1:200).

### Peptide ELISA.

A total of 45 peptides covering all parts of CaSR-ECD were synthesized: 1: 1–20; 2: 21–31; 3: 32–43; 4: 44–58; 5: 56–70; 6: 70–79; 7: 79–95; 8: 96–110; 9: 108–122; 10: 123–137; 11: 138–150; 12: 151–164; 13: 165–179; 14: 177–191; 15: 187–203; 16: 204–213; 17: 214–238; 18: 239–255; 19: 256–266; 20: 267–281; 21: 282–292; 22: 293–307; 23: 308–314; 24: 314–323; 25: 322–329; 26: 330–343; 27: 344–358; 28: 359–373; 29: 374–391; 30: 392–405; 31: 406–420; 32: 419–428; 33: 428–440; 34: 441–455; 35: 456–470; 36: 471–483; 37: 484–493; 38: 494–508; 39: 506–520; 40: 519–532; 41: 533–546; 42: 547–561; 43: 559–573; 44: 571–585; and 45: 586–603 (Sigma-Aldrich, GenScript). Peptides consisting of extracellular loops 1 (peptide 46: 667–686), 2 (peptide 47: 724–773), and 3 (peptide 48: 824–841) were also synthesized (Figure 8, A and B). One microgram of each peptide was dissolved in peptide-binding buffer (50 mM carbonate-bicarbonate buffer, pH 9.6) and incubated at 37°C for 1 hour in 96-well plates, followed by washing 3 times with PBS-Tween 0.05%. After blocking with 1% BSA/PBS-Tween 0.1% at 37°C for 1 hour, cells were incubated by each serum (1:500) or anti-CaSR [clone 5C10, ADD] (catalog ab19347, Abcam) (1:8,000) in 1% BSA/PBS-Tween 0.1% at room temperature for 1 hour. Following washing 3 times with PBS-Tween 0.05%, cells were incubated by antihuman antibody (catalog 074-1002, Kirkegaard and Perry Laboratories) (1:5,000) for wells reacted by sera or antimouse antibody (catalog 074-1804, Kirkegaard and Perry Laboratories) (1:5,000) for wells reacted by anti-CaSR at room temperature for 1 hour. After washing 3 times with PBS-Tween 0.05%, the cells were treated with substrate (*o*-phenylenediamine dihydrochloride, MilliporeSigma) for 5 minutes at room temperature. This reaction was stopped by the addition of an equivalent volume of 2.5 N HCl and the absorption levels were read at 492 nm using a plate reader.

### Statistics.

For statistical analysis, 2-sided Student’s *t* test (for 2 comparisons) and 2-sided Tukey’s multiple comparison procedures (for multiple comparisons) were used. All analyses were performed using Prism 8 software. Averaged data from more than 3 independent experiments are shown, and error bars represent the SEM unless otherwise stated. A value of *P* < 0.05 was considered significant for all analyses. *P < 0.05; **P < 0.01; ***P < 0.001.

### Study approval.

This study was performed under approval of the Institutional Review Board of the University of Tokyo (approval number G1067), and written informed consent was received from our patients with AHH prior to inclusion in this study.

## Author contributions

NM, YH, HH, and JS performed the experiments. KA, T Ito, HY, and AM clinically diagnosed the patients as having AHH and followed patients. MN, KM, NM, and T Iiri analyzed the data. MN and T Iiri wrote the paper, and all authors discussed the manuscript.

## Supplementary Material

Supplemental data

## Figures and Tables

**Table 1:**
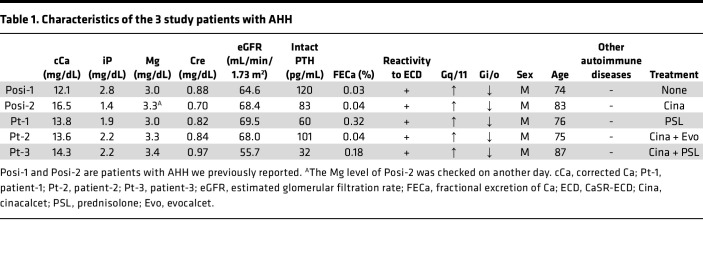
Characteristics of the 3 study patients with AHH

## Version 1 09/13/2022

In-Press Preview

## Version 2 10/24/2022

Electronic publication
