# Supplementary material for: Successful prednisolone or calcimimetic treatment of acquired hypocalciuric hypercalcemia caused by biased allosteric CaSR autoantibodies
Source: JCI Insight. 2022 Oct 24;7(20):e156742. doi: 10.1172/jci.insight.156742 (PMC9714797; doi:10.1172/jci.insight.156742)
Supplement: Supplemental data [file jciinsight-7-156742-s056.pdf]

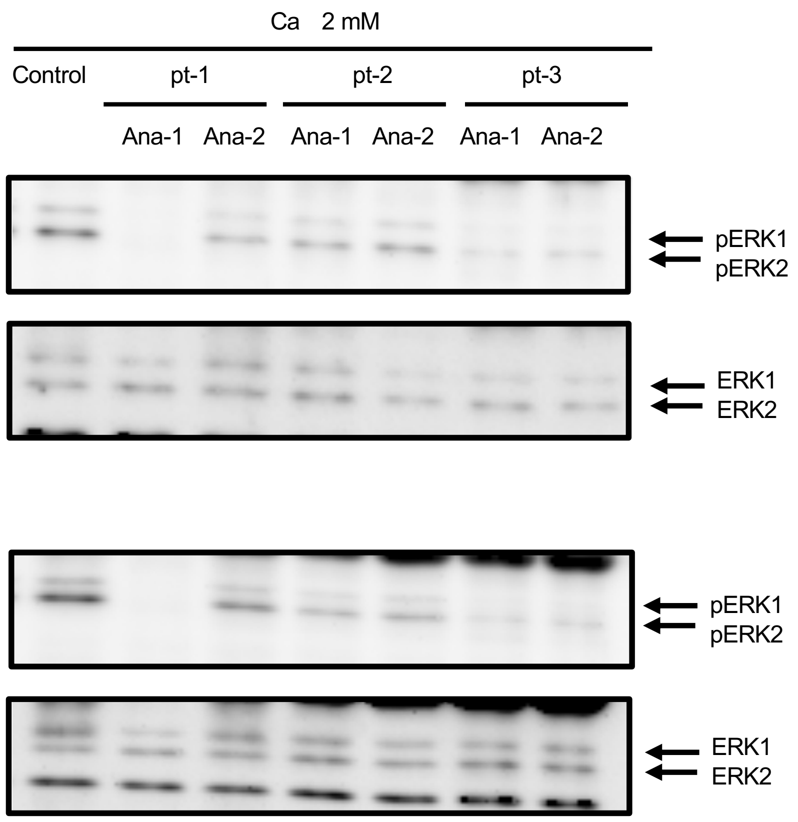


**Supplementary Figure 1. Immunoblotting of pERK1/2 and ERK1/2 in cells exposed to 2.0 mM Ca with the co-administration of the AHH patients' IgG at two timepoints**

HEK293 cells stably expressing human CaSR were exposed to 2 mg/dL of AHH patients' IgG, and stimulated with 2.0 mM Ca. The phosphorylation of ERK1/2 was detected by immunoblotting, and each ERK1/2 was detected by reblotting of the same membrane.
